# Effect of Fat Content on Rice Taste Quality through Transcriptome Analysis

**DOI:** 10.3390/genes15010081

**Published:** 2024-01-09

**Authors:** Jie Guo, Xinqiao Zhou, Dagang Chen, Ke Chen, Chanjuan Ye, Juan Liu, Shaolong Liu, Youding Chen, Guorong Chen, Chuanguang Liu

**Affiliations:** 1 Rice Research Institute, Guangdong Academy of Agricultural Sciences, Guangzhou 510640, China; guojie@gdaas.cn (J.G.); zhouxq@gdaas.cn (X.Z.); chenguorong@gdaas.cn (G.C.); 2 Key Laboratory of Genetics and Breeding of High Quality Rice in Southern China (Co-Construction by Ministry and Province), Ministry of Agriculture and Rural Affairs, Guangzhou 510640, China; 3 Guangdong Key Laboratory of New Technology in Rice Breeding, Guangzhou 510640, China; 4 Guangdong Rice Engineering Laboratory, Guangzhou 510640, China

**Keywords:** rice quality, fatty acids, transcriptome, taste quality

## Abstract

Rice is an important crop in the word, and fat is one of the main important nutrient components of rice. The lipid content and fatty acid composition of grains significantly influences the quality of rice. In this study, 94 homozygous recombination inbred lines (RILs) were developed and the crude fat content of them displayed a normal distribution ranging from 0.44% to 2.62%. Based on their taste quality, a positive association between fat content and eating quality was revealed. Then, two lines (FH and FL) were selected with similar agronomic characteristics and different lipid content and taste quality for RNA sequencing analysis, and a total of 619 differentiable expressed genes were detected, primarily enriched in metabolic pathways such as starch and sucrose metabolism, fatty acid metabolism, and amino acid metabolism. The expression of two genes related to fatty acid synthesis and elongation was significantly up-regulated, while the expression of three genes related to fatty acid degradation was significantly down-regulated in FH grains. By using liquid chromatography, the relative levels of palmitic acid and oleic acid were discovered significantly higher in FH grains. Additionally, the comparative genomic analysis was conducted to visualize genomic differences of five genes. Ultimately, two genes (*Os07g0417200* and *Os12g0102100)* were selected to be the key gene to affect the lipid metabolism, especially for the synthesis of unsaturated fatty acids, significantly changing the eating quality of rice. These results provide a theoretical basis for improving the taste quality of rice.

## 1. Introduction

Rice is an important food crop. With the improvement of people’s living standards, the demand for rice quality has been on the rise. The quality traits of rice mainly include processing quality, appearance quality, cooking and taste quality, nutritional quality, and aroma. While the improvements in appearance, cooking, and aroma quality could be achieved through techniques like molecular marker-assisted selection and hybridization [1,2,3], enhancing the taste quality of rice remained a significant challenge in rice breeding. Fat is one of the three major nutrients, despite its low content. And, rice primarily consists of high-quality unsaturated fatty acids that contribute to its nutritional value [4,5]. Additionally, fat content could significantly impact the taste and nutritional quality of rice [6,7]. Therefore, it was necessary to investigate the influence of fat content in rice on its taste and nutritional quality.

The lipid content of rice primarily existed in two forms: bound lipids (starch lipids) and free lipids (non-starch lipids). In brown rice, the content was approximately 3%, while in polished rice, it is around 0.8% [7]. Moreover, the lipid content would decrease with the improvement of milling accuracy [8]. Additionally, the lipid content was influenced by factors such as variety, growing environment, cultivation conditions, storage time, and processing methods [9,10,11,12]. Non-starch lipids mainly consisted of oleic acid, linoleic acid, and palmitic acid, of which unsaturated fatty acids account for a relatively large proportion, with high nutritional value. Starch lipids primarily included palmitic acid and linoleic acid, forming complexes with straight-chain starch in the endosperm, thereby affecting starch digestion characteristics [13,14].The content of starch and non-starch lipids significantly impacted the appearance quality and cooking taste quality of rice. Within a certain range, there was a notable positive correlation between lipid content and taste quality in rice. Higher lipid content was associated with improved appearance quality and taste quality, leading to characteristics such as distinct oiliness in rice grains and good palatability [13,15,16,17,18]. Lipids also affect starch digestion, displaying a significant positive correlation with resistant starch content and a negative correlation with starch digestion characteristics and glycemic index [19]. Rice needs to be stored under low-temperature conditions to prevent environmental influences during storage. Non-starch lipids, particularly unsaturated fatty acids, were susceptible to oxidative acid transformation leading to quality degradation of rice [20,21]. But, more serious quality degradation had not been found in high-fat rice [22]. Consequently, a rational increase in rice lipid content and fatty acid composition played a vital role in enhancing rice taste quality and nutritional value.

The synthesis and metabolism of lipids in rice are complex processes regulated by multiple loci. While many Quantitative Trait Loci (QTL) related to lipid synthesis, metabolism, or genetic improvement have been identified [23,24], only little lipid-related genes were cloned. Based on whole-genome association analysis, Zhou et al. [25] cloned four genes and proposed a biosynthetic pathway for lipids in rice, where *qPAL6* controls the synthesis of fatty acid C16:0, *LIN6* influences the synthesis of unsaturated fatty acids C18:1 and C18:2, *qMYR2* impacts the synthesis of saturated fatty acids C14:0 and C16:0, and qARA6 affects the synthesis of fatty acids in rice. *qFC6*, a major quantitative trait locus was cloned, and negatively regulated crude fat content and rice quality [26].

Moreover, unsaturated fatty acids have been shown to play a fundamental role in health. The genes encoding for desaturases have been identified as being responsible for the synthesis of unsaturated fatty acids [27,28]. There are 20 fatty acid desaturase genes in rice according to bioinformatic analysis [29]. The synthesis of saturated fatty acids to unsaturated fatty acids in rice is mainly catalyzed by these desaturases. Liu et al. [30] confirmed that the expression of the Fatty Acid Desaturase 3 (*OsFAD3*) gene can convert linoleic acid into linolenic acid. Zaplin et al. [31] demonstrated the crucial role of the Fatty Acid Desaturase 2 (*FAD2*) gene in the conversion of oleic acid (C18:1) to linoleic acid (C18:2) in rice grains. Then, using gene editing technology to eliminate *OsFAD2-1*, showing that oleic acid content increased by two times compared with the wild type [32], providing a method to increase the relative content of unsaturated fatty acids in rice grains without changing the main agronomic traits.

Nonetheless, the molecular mechanisms underlying the influence of lipid content on rice taste quality remain unclear. In this study, we developed recombination inbred lines generated from materials with distinct rice quality traits to analyze the correlation between rice fat content and taste quality. Furthermore, the RNA-sequencing (RNA-seq) was performed to obtain the expression of genes related to fat acid synthesis in FH line with high taste quality. Then, we investigated the component of fatty acids in FH and FL lines. Taking these together, we revealed the effect of fat content on rice taste quality. Hence we hypothesized that our study would provide a foundational understanding for the molecular design and breeding of high-fat, high-quality rice varieties.

## 2. Materials and Methods

### 2.1. Plant Materials

Three *indica* rice varieties, Huangguanglizhan (HGXZ), Xinhuangzhan (XHZ), and Xiangyaxiangzhan (XYXZ) were provided by the Rice Research Institute of Guangdong Academy of Agricultural Sciences. In this study, the HGLZ was crossed with XHZ to develop a material with high yield and disease resistance in the F_3_ generation by molecular marker selection and field resistance identification. Subsequently, this material was crossed with the Simiao rice variety XYXZ. The single seed descend method was used to develop recombination inbred lines (RILs). In the F_2_ population, 94 plants with slender grain types were gained from F_1_ hybrids. Then, only one seed from each individual was selected to plant per generation, with multi-generation self-cross until the F_5_ generation, and a population of 94 recombination inbred lines was developed.

### 2.2. Main Agronomic Traits

Twenty plants from the middle two rows were examined for each trait. Plant height was measured as the length from the base of the stem on the ground to the top of the rice panicle (excluding awns), with the average height calculated for each material. The number of effective tillers per single plant was recorded and averaged. After sun drying, traits such as panicle length, grain length, and grain width were measured on each panicle with three replicates.

### 2.3. Determination of Protein, Fat, and Apparent Amylose Contents of Rice Grains

The rice grains were dehulled, polished, and pulverized, passed through a 100-mesh aperture. The apparent amylose content was determined with the standard iodine colorimetric method according to ISO 6647–2 [33,34]. The absorbance was at 620 nm, and the quantification was achieved with samples comprising four levels (1.5%, 10.4%, 16.2%, and 26.5%) provided by the China National Rice Research Institute. For measuring rice crude protein content, sulfuric acid digestion followed by the Kjeldahl method was employed, with a conversion factor of 5.95 [35]. The crude fat content in rice was determined using the Soxhlet extraction method, and was also determined by an automatic Soxlet extraction system (FOSS SOXTEC2050, Copenhagen, Denmark),produced by the FOSS company according to the instructions. Petroleum ether with boiling point of 30~60 °C was used as extraction solvent. This was repeated 3 times for each sample. Non-starch lipid content in rice was determined using the previously reported chloroform-methanol extraction method [36].

### 2.4. Determination of Rice Fatty Acid Components

For the determination of rice fatty acid components, grain samples at 21 days after flowering were collected from two materials, FH and FL. Three replicates were collected as biological replicates and were flash-frozen in liquid nitrogen. The samples were then stored in a −80 °C freezer and sent to Baimaike Biological Technology Company Limited.(Beijing, China), for sample processing and extraction. The data acquisition instrument system primarily consisted of Ultra Performance Liquid Chromatography (UPLC) (SHIMADZU Nexera X2, https://www.shimadzu.com.cn/ accessed on 8 May 2021) and Tandem Mass Spectrometry (MS/MS) (Applied Biosystems QTRAP, http://www.appliedbiosystems.com.cn/ accessed on 8 May 2021). Samples were injected onto a Xselect HSS T3 (2.1 × 150 mm, 2.5 μm) using a 20 min linear gradient at a flow rate of 0.4 mL/min for the positive/negative polarity mode. The eluents were eluent A (0.1% Formic acid-water) and eluent B (0.1% Formic acid-acetonitrile). The effluent was alternatively connected to an ESI-triple quadrupole-linear ion trap (QTRAP)-MS (ABI, Wilmington, DE, USA). The mass spectrometer was operated in positive polarity mode with curtain gas of 35 psi, collision gas of medium, ion spray voltage of 5500 V, temperature of 550 °C, ion source gas of 1:60, and ion source gas of 2:60. Based on a local metabolic database, qualitative and quantitative analysis of major lipid metabolites in the samples was performed using mass spectrometry. Prior to analysis, data were normalized using internal standards, with each metabolite’s peak area divided by the peak area of the internal standard metabolite.

### 2.5. Determination of Taste Value

Using XYXZ as a reference, under identical conditions, we soaked rice for 30 min, steamed it in a rice cooker for 40 min, the ratio of rice to water was 1:1.2, and kept it warm for 10 min. Then, we determined the taste value of rice by using a rice taste analyzer (Satake, Hiroshima, Japan). Ratings of 90 and above were classified as Grade I, ratings between 85 and 90 as Grade II, and ratings below 85 as Grade III.

### 2.6. Sample Collection and Total RNA Extraction

Grain samples with 14 days of grain filling from two lines, FH and FL, were selected, with three biological replicates each. After flash freezing in liquid nitrogen, the samples were stored at −80 °C. RNA extraction, quality control, library construction, and Illumina Novaseq™ 6000 sequencing were outsourced to Baimaike Biological Technology Company Limited (Beijing, China). The RNA concentration and purity was measured using NanoDrop 2000 (Thermo Fisher Scientific, Wilmington, DE, USA). RNA integrity was assessed using the RNA Nano 6000 Assay Kit of the Agilent Bioanalyzer 2100 system (Agilent Technologies, Palo Alto, CA, USA).

### 2.7. Library Construction for Transcriptome Sequencing

A total amount of 1 μg RNA per sample was used as input material for the RNA sample preparations. Sequencing libraries were generated using NEBNext UltraTM RNA Library Prep Kit for Illumina (NEB, Ipswich, MA, USA) following manufacturer’s recommendations, and index codes were added to attribute sequences to each sample. The original read data (raw reads) were filtered. In this paper, the clean data (clean reads) were obtained by removing reads containing an adapter, reads containing ploy-N, and low-quality reads from raw data. The Q20, Q30, GC-content and sequence duplication level of the clean data were calculated (Table S2). Then, these clean reads were mapped to the reference genome sequence which was downloaded from RAP-DB (http://rapdb.dna.affrc.go.jp/, accessed on 8 July 2021). Only reads with a perfect match or one mismatch were further analyzed and annotated based on the reference genome. HISAT2 tools soft (v2.0.4) were used to map with reference genome. Expression level analysis was conducted for all genes that were successfully aligned.

### 2.8. DEG Identification and Analysis

DEGseq2 software (v1.4.5) was employed to compare the gene expression levels between FH and FL, which exhibited different fat contents but the same grain-filling time. Significant differential analysis was performed, defining genes with a fold change ≥2 or ≤0.5 and a *p*-value < 0.05 as differentially expressed genes. These genes were annotated in the Gene Ontology (GO) (http://www.geneontology.org/page/download-go-annotations/, accessed on 12 July 2021) and Kyoto Encyclopedia of Genes and Genomes (KEGG) databases (www.genome.jp/kegg/, accessed on 12 July 2021) to obtain functional annotations and related metabolic pathway information for the differentially expressed genes. The GO enrichment analysis was implemented by the GOseq R packages based Wallenius non-central hyper-geometric distribution [37], and the KOBAS software (v3.0) [38] was used to test the statistical enrichment of differential expression genes in KEGG pathways.

### 2.9. Real-Time Quantitative PCR Analysis

To validate the RNA-Seq results, several genes with differential expression patterns were confirmed using real-time quantitative PCR. Total RNA was extracted from grains after 14 days of grain filling using the TRIzol reagent (Invitrogen, Carlsbad, CA, USA). The integrity and concentration of the extracted RNA were detected by agarose gel electrophoresis and scanning with a microspectrophotometer (NanoDrop 2000). Reverse transcription and cDNA synthesis was performed using ReverTra Ace qPCR RT Master Mix with gDNA Remover (Toyobo, Osaka, Japan) from 200 ng of total RNA. qRT-PCR was performed following the kits instructions, the first CDNA chain obtained by reverse transcription of 1 μg total RNA was used as the template, and the Fast Start Universal SYBR Green Master Mix with ROX (Roche, Basel, Switzerland) was used in a 20 μL reaction system. Then, the amplification process was completed by an ABI 7500 real-time quantitative PCR instrument (Applied Biosystems, Waltham, MA, USA). The reactions were repeated three times, and the 2^−ΔΔCt^ method was employed to determine the relative expression levels of the target genes [39]. The reference gene used was *Actin*, and some primers were sourced from references [40,41] and listed in Table S10.

### 2.10. Data Analysis

Data collection and processing were performed using Microsoft Excel and Origin 8.0. Significance analysis was conducted using SPSS 16.0 software. Data are presented as mean ± standard deviation. Multiple comparisons of means were carried out using the Least Significant Difference (LSD) method, and results were denoted using letter labeling. The same letter indicates no significant difference, while different lowercase letters represent significant differences between varieties (*p* < 0.05). The *t*-test was used to detect significant differences in rice quality indicators between the two materials or types, with * indicating *p* < 0.05 (significant difference) and ** indicating *p* < 0.01 (highly significant difference).

## 3. Results

### 3.1. Rice Quality of Materials

In this study, three rice varieties were selected from the Guangdong core germplasm resource bank: XHZ and HGLZ (which were with high-yielding and disease-resistant traits) and XYXZ (a Simiao rice variety with faint scent and tastiness). These three materials exhibit distinct rice quality characteristics. Multiple comparisons revealed that XHZ had the highest amylose content and protein content, while it had the lowest crude fat content and non-starch lipid content. In contrast, HGLZ possessed the lowest amylose content but the highest non-starch lipid content. XYXZ had the highest crude fat content among the rice varieties, but its amylose content was significantly higher than that of HGLZ (Table 1).

### 3.2. Construction of Recombination Inbred Lines and Its Eating Quality

In this study, XYXZ was crossed with the high-yielding and disease-resistant intermediate materials HGYZ/XHZ. Then, we selected single plants with slender grain types and self-pollinated them to obtain 94 recombination inbred lines (RILs) in F_5_. Subsequently, we analyzed the rice quality of these RILs. The results indicated that the amylose content of the 94 RILs ranged from 13.50% to 19.31%, protein content ranged from 5.30% to 7.70%, the crude fat content ranged from 0.44% to 2.62%, and non-starch lipid content ranged from 0.16% to 0.38%. Based on taste analyzer evaluations of rice aroma, appearance, palatability, taste, and cold rice texture, these RILs were classified into three categories: Grade I (above 90 points), Grade II (between 85 and 90 points), and Grade III (below 85 points). Among the RILs, 17 materials achieved Grade I, 23 achieved Grade II, and 54 were categorized as Grade III in taste evaluations (Table S1).

**Figure 1:**
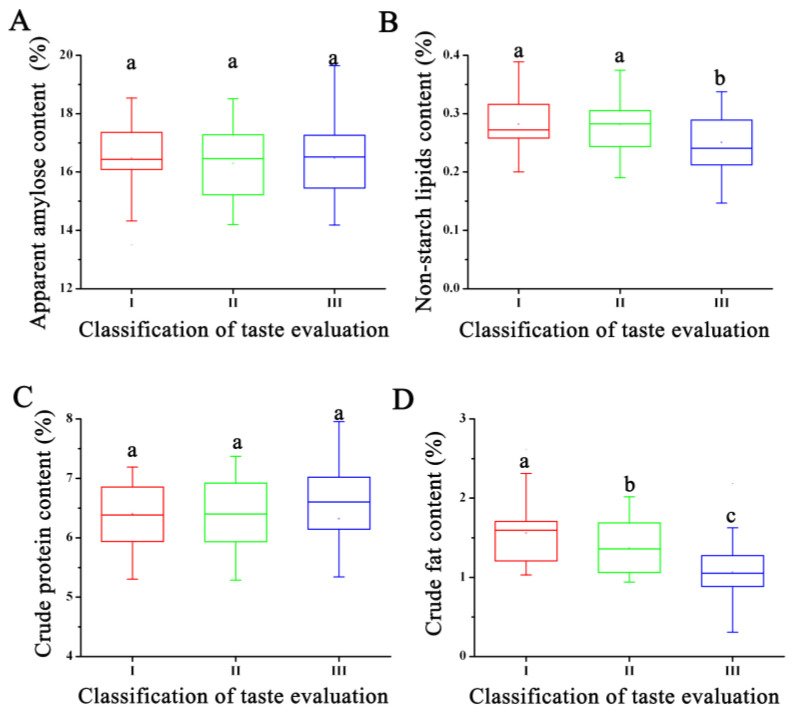
The difference analysis of the nutritional quality and eating quality of rice variety. (**A**) The apparent amylose content of rice with different taste quality. (**B**) The non-starch lipids content of rice with different taste quality. (**C**) The crude protein content of rice with different taste quality. (**D**) The crude fat content of rice with different taste quality. Note: The least significant difference (LSD) method is used to compare the means between different recombinant inbred lines with different taste evaluation. Different letters within the same column indicate significance at 0.05 probability level.

Further analysis of the rice quality characteristics of RILs in different categories revealed that there were no significant differences in amylose and protein content among the three rice quality grades (Figure 1). However, Grade III lines, which had lower taste quality, exhibited the lowest crude fat and non-starch lipid content. It also had the highest variation in amylose and protein content. In contrast, Grade I rice, which had the highest quality, had the highest crude fat and non-starch lipid content and the smallest variation in amylose and protein content. Variance analysis results indicated that the fat content of rice maybe influence the rice taste quality.

### 3.3. Correlation Analysis of Crude Fat Content and Other Eating Qualities in 94 RILs

**Figure 2:**
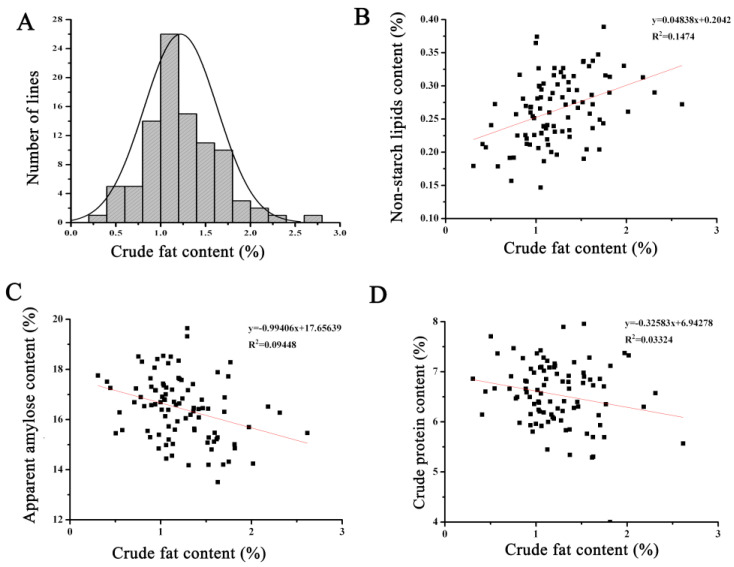
The correlation analysis of crude fat content and other nutritional quality in recombinant inbred lines. (**A**) Normal distribution of crude fat content in recombinant inbred lines. (**B**) The correlation analysis of crude fat content and non-starch lipids content. (**C**) The correlation analysis of crude fat content and apparent amylase content. (**D**) The correlation analysis of crude fat content and crude protein content.

The crude fat content of the 94 homozygous RILs displayed a normal distribution, indicating that this trait follows a quantitative pattern and is influenced by minor-effect polygenes (Figure 2A). Correlation analysis between crude fat content and other quality traits revealed a negative correlation between rice crude fat content and protein content as well as amylose content, although not statistically significant. Fat content comprises both starch lipids and non-starch lipids, and the crude fat content was positively correlated with non-starch lipid content, albeit without statistical significance. Even in materials with the highest fat content, the non-starch lipid content only reached 0.27% (Figure 2). Thus, increasing fat content may not necessarily lead to a significant increase in non-starch lipid content.

### 3.4. Phenotyping of Two Lines with Different Taste Quality

**Figure 3:**
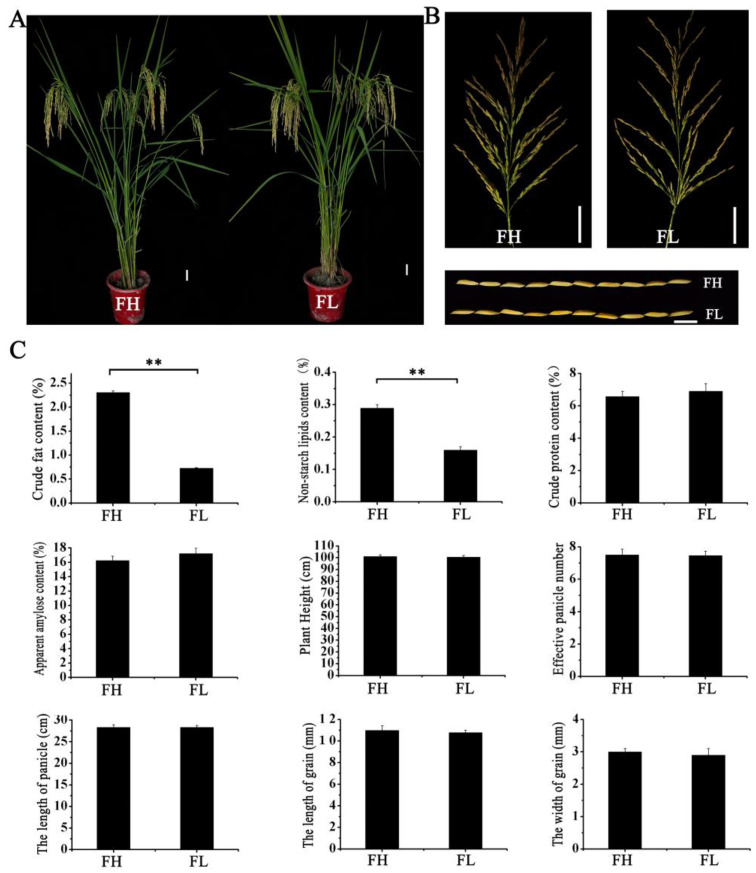
The agronomic and rice quality traits of FH and FL. (**A**) Plant type. (**B**) Spikelet type and grain type. (**C**) Main agronomic traits and rice quality traits. Note: (1) The two-sided *t*-test was used to test the difference significance between FH and FL on agronomic and rice quality traits. ** indicates significance at the 0.01 probability level.

Fat content was closely associated with the cooking quality of rice. In this study, two materials were selected from the RILs: FH (best taste quality in Grade I) and FL (the taste quality in Grade III). Both materials possessed aroma traits. An analysis of their main agronomic traits revealed no significant differences in plant height, effective panicles, panicle length, grain length, and grain width between FH and FL. Additionally, the length-to-width ratio of their grains exceeded 3.6 (Figure 3). Subsequently, nutritional quality parameters of two lines were measured. The results indicated that FH and FL exhibited no significant differences in amylose and protein content. However, the crude fat content and non-starch lipid content of the FH material were significantly higher than those of the FL material (Figure 3). This suggests that the main factor affecting the difference in taste quality between these two lines may be the varying fat content in the rice endosperm.

### 3.5. Analysis of Differentially Expressed Genes between FL and FH Lines

**Figure 4:**
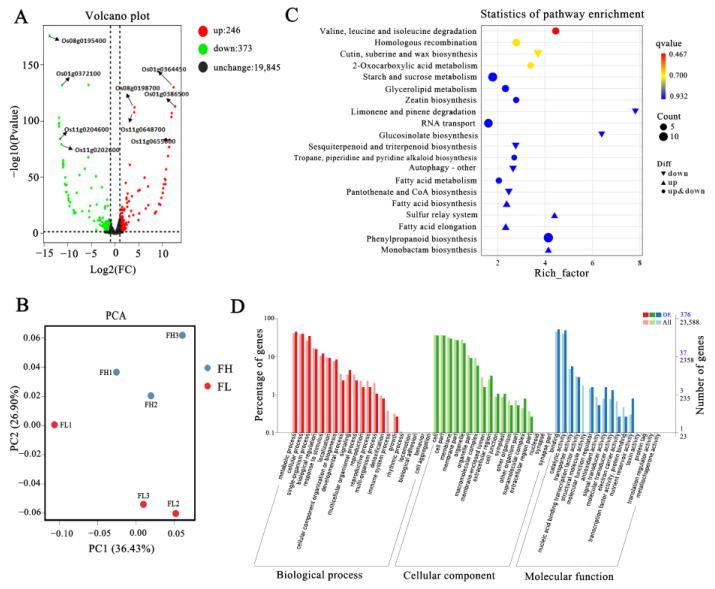
Transcriptome analysis of rice quality changes between FH and FL materials. (**A**) Volcano map of differentially expressed genes. (**B**) PCA score plot of two grains in FH and FL lines. (**C**) Bubble chart of KEGG enrichment analysis of differently expressed genes. (**D**) GO enrichment and GO terms of RNA-seq data.

To further explore the molecular mechanisms influencing rice taste quality, transcriptome analysis was conducted using grain samples, 14 days after anthesis, from two lines FH and FL, with three replicates each. After removing low-quality and adapter-contaminated sequences from the raw data, the Q20% (sequencing error rate less than 0.01) of each sample exceeded 98%, and the Q30% exceeded 94%. The GC content ranged from 50.05% to 50.98% (Table S2). Using japonica Nipponbare as the reference genome, the alignment rates of each sample exceeded 93%, with paired-end alignment rates exceeding 88%. These results indicate the high accuracy of the sequencing data. In addition, in order to gain deeper insights into the expression profiles of differentially expressed genes in each sample, PCA analysis was performed. The three replicates of FH and FL clustered separately, but the distances between them were close, suggesting that the expression profiles of most genes in FH and FL were similar, possibly due to their high genetic similarity (Figure 4B). The filtered data were subsequently used for gene expression analysis, differential gene screening, and bioinformatics analysis.

A volcano plot revealed that a total of 619 differentiable expressed genes were identified compared to FL, with 373 genes (60.25%) showing down-regulation and 246 genes (39.74%) displaying up-regulation (Figure 4A, Table S3). To better explore these DEGs enrichment, we performed GO analysis (Figure 4D). In the Biological Process (BP), the main DEGs were associated with metabolic process, cellular process, single-organism process, and biological regulation; while the distribution of DEGs in Cellular Component (CC) were focusing on cell, cell part, membrane, and membrane part; in the Molecular Function (MF), the DEGs mainly associated with binding, catalytic activity, transporter activity, and nucleic acid binding transcription factor activity (Figure 4D). In addition, we also exhibited the DEGs in the KEGG annotation. The top 20 terms used for enriching those detected DEGs were generated and then clustered into organismal systems, metabolism, genetic information processing and environmental information processing. And, they were significantly enriched in pathways related to starch and sucrose metabolism, fatty acid metabolism, fatty acid biosynthesis, amino acid metabolism, phenylpropanoid biosynthesis, branched-chain amino acid degradation (valine, leucine, and isoleucine), and RNA transport (Figure 4C). This suggested that the different fat content of endosperm triggered significant changes in secondary metabolites and the expression of fatty acid metabolism genes, revealing these metabolism processes may cause changes in the taste quality of rice.

### 3.6. Underlying Regulation of Fat Content Influenced Rice Taste Quality Improvement

**Figure 5:**
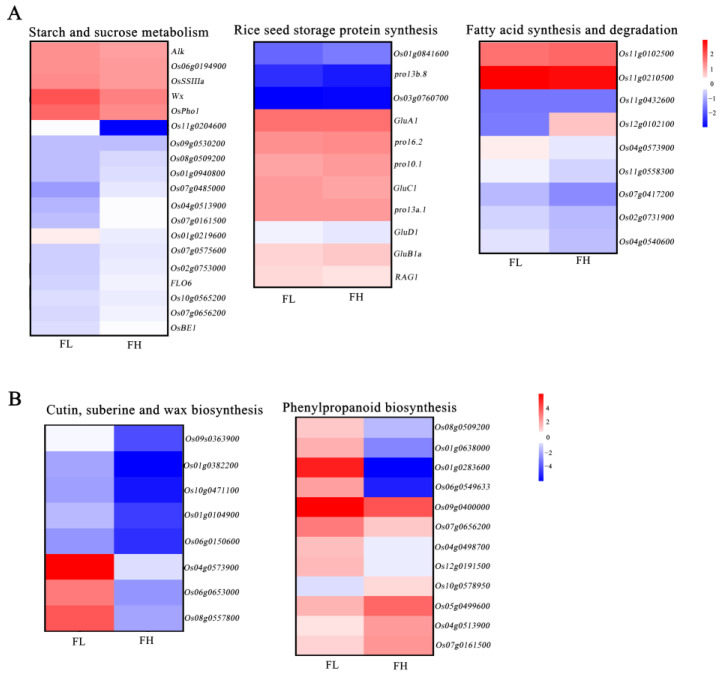
Analysis of genes differentially expressed in FH material compared with FL material. (**A**) Heat map of expression change of genes selected in the starch and sucrose, storage protein, and fatty acid metabolism. (**B**) Heat map of expression change of genes selected in the DEGs. The color in each frame represents the level of expression change based on the log2 fold.

The amylose content, protein content, and fat content of rice were the key factors that were suggested to determine cooking quality. Thus, we selected genes related to starch synthesis, protein synthesis, and fatty acid synthesis from the pool of differentiable expressed genes. Cluster analysis of their expression levels revealed that in the starch synthesis pathway, FH, known for its superior rice quality, the major genes displayed both up-regulated and down-regulated expressions. For instance, the gene encoding granule-bound starch synthase *Wx* [42], which was the major gene synthesizing amylose in rice endosperm, was down-regulated in FH. While genes encoding starch synthase II (*Alk* [43]), soluble starch synthase (*OsSSIIIa* [41]), branching enzyme (*OsBEIIb* [44]), and genes associated with chalkiness formation, such as *FLO6* [40], were up-regulated in FH (Figure 5A, Table S4). But, we analyzed that the *Wx* gene of FH and FL materials was the same allele by using functional markers, which suggests different expressions of the *Wx* may be caused by the changes in its upstream genes in these two materials. But, these genes collectively influenced starch synthesis in rice grains, ultimately resulting in no significant difference in grain starch content between the two materials. Regarding the process of protein synthesis, genes encoding storage proteins, including *GluA*, *GluB*, *GluC*, and *GluD* [45], did not exhibit significant differences in expression between FH and FL (Figure 5A, Table S5). This suggests that these genes may not play a substantial role in regulating the variation in rice quality between the two recombinant inbred lines.

Additionally, the genes related to fatty acid synthesis and elongation, such as *Os11g0102500* and *Os11g0210500*, displayed significantly increased expression. In contrast, genes associated with fatty acid degradation, including *Os04g0540600*, *Os07g0417200*, and *Os04g0573900*, were notably down-regulated. The gene *Os12g0102100*, encoding a dehydrogenase likely involved in desaturating saturated fatty acids into unsaturated fatty acids, showed significantly increased expression in FH grains during grain filling (Figure 5A, Table S6). Furthermore, we found that the majority of genes related to cuntin, suberine, wax biosynthesis, and phenyloropanioid biosynthesis showed down-regulation in FH (Figure 5B, Tables S7 and S8). This process may be involved in regulating the synthesis of related metabolites in the endosperm. All these findings suggested that the interplay among these differentiable expressed genes may contribute to the differences in fatty acid content between the two materials, ultimately resulting in differences in taste quality between FH and FL.

**Figure 6:**
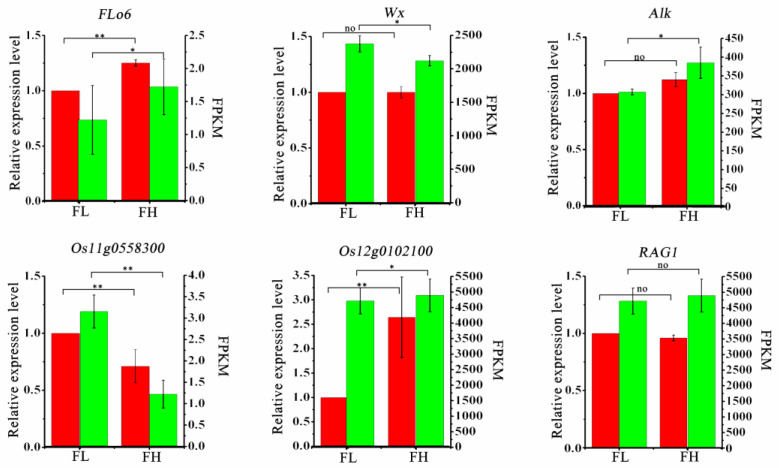
qRT-PCR analysis of differently expressed genes of rice quality. Note: The data in the red bars of the graphs are the mean ± standard deviation of gene expression from three biological replicates, with *actin* used as an internal reference for the homogenization calculations, in which the gene expression of the FL material was normalized to 1, and the vertical coordinates are on the left. The data in the green bar graph are FRKM values from transcriptome analysis, with vertical coordinates on the right. The two-sided *t*-test was used to test the difference significance in gene expression or transcriptome FRKM values between the two recombinant inbred lines, FH and FL, and “*” indicated the significance at 0.05 probability level (*p* < 0.05), “**” indicated the significance at 0.01 probability level (*p* < 0.01), and “no” indicated that the difference was not significant.

To validate the authenticity of the transcriptome sequencing results, we selected six genes (*Flo6*, *Alk*, *Wx*, *RAG1*, *Os12g0102100*, *Os11g0558300*) related to rice quality, with Actin used as the internal reference gene. We performed qRT-PCR validation analysis of their transcription levels in the grains of the FH and FL lines during the filling stage. The fragments per kilobase of transcript per million fragments mapped (FPKM) is an indicator for measuring the expression levels of transcripts or genes. The results showed that the trends in the FPKM values of these genes were generally consistent with the results obtained from qRT-PCR validation (Figure 6), indicating the reliability of the transcriptome sequencing data.

### 3.7. The Sequence Variation Analysis of Five Genes Related to Fatty Acid Synthesis

**Figure 7:**
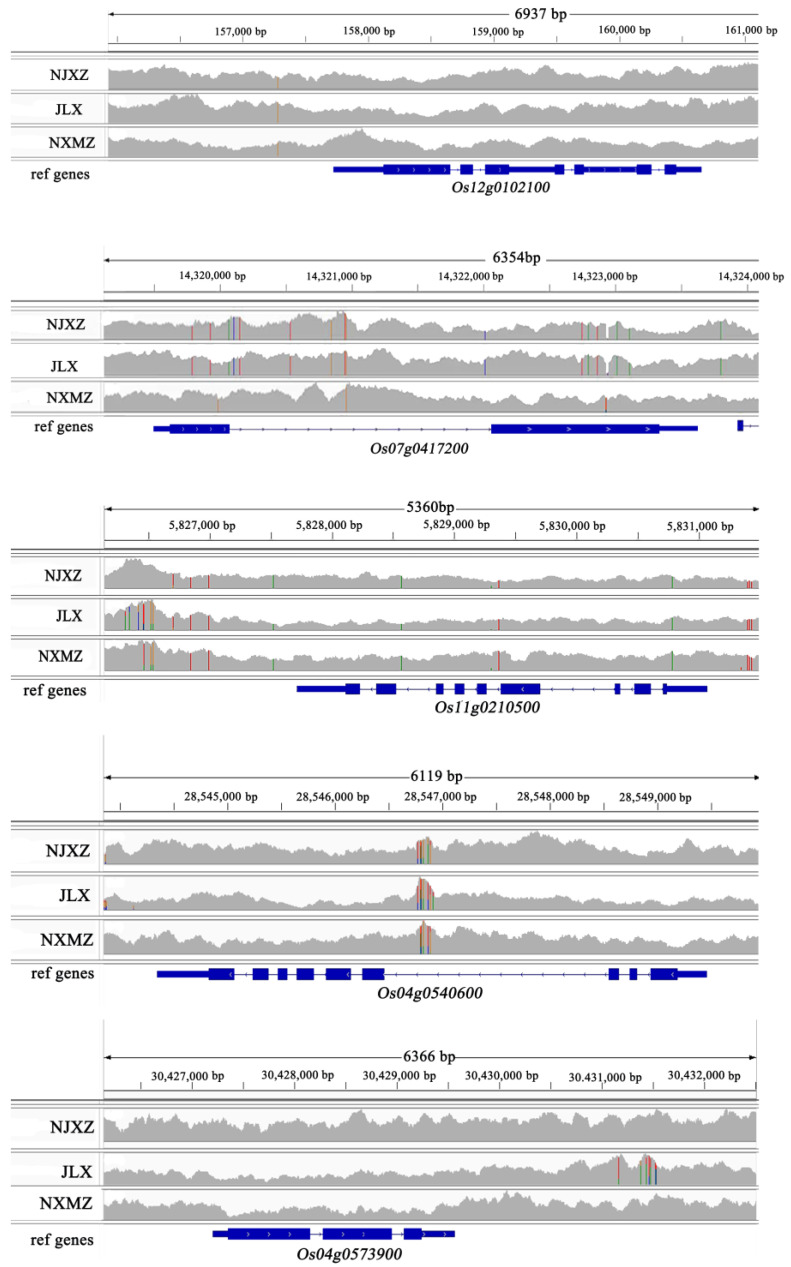
The haplotype of five fatty anabolism-related genes among three rice cultivars. The special color in the bam file showed the variation positions in target genes in comparison to reference genome.

In this study, the FH material which exhibited favorable appearance and taste qualities among the recombinant inbred lines was named Jiulixiang (JLX). Using Zhenshan 97 as the reference genome, high-quality Guangdong Simiao rice variety Nanjing Xiangzhan (NJXZ) and high-yield rice variety Nanxiumeizhan (NXMZ) were chosen as controls. Comparative genomic analysis was conducted using the Integrative Genomics Viewer (IGV) to visualize genomic differences related to lipid synthesis metabolism in JLX compared to the reference genome. The results revealed that, in comparison to the reference genome, three genes *Os12g0102100*, *Os11g0210500*, *Os04g0573900* in JLX, NJXZ, and NXMZ exhibited consistent variations in gene regions. But, a SNP difference was found in the promoter region of the gene *Os12g0102100*, which encodes a dehydrogenase. The gene *Os04g0540600*, encoding aldehyde dehydrogenase, showed multiple SNP sites in the intron region between JXL, NJXZ, and NXMZ. Additionally, JLX and NJXZ displayed extensive variations in both the exon and intron regions of the gene *Os07g0417200*, while the haplotype of NXMZ remained consistent with the reference genome sequence. This gene encodes a fatty acid desaturase, impacting fatty acid synthesis metabolism (Figure 7).

### 3.8. Analysis of Lipid Composition

Metabolites serve as the foundation of an organism’s phenotype, aiding in a more intuitive and effective understanding of biological processes and their mechanisms. In this study, two materials, FH and FL, with different quality grades were sampled at the 21st day of grain filling, with three replicates each. The relative levels of lipid-related metabolites were determined.

**Figure 8:**
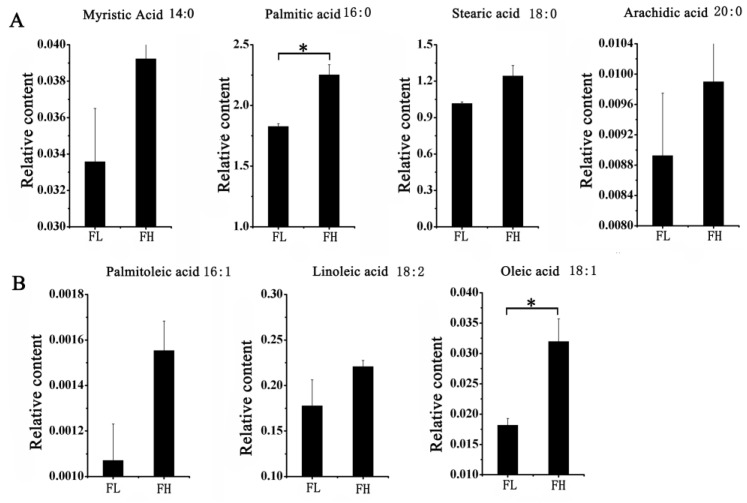
Comparison of the relative amount of main fatty acids in FH and FL of rice. (**A**) The relative amount of four saturated fatty acid in FH and FL. (**B**) The relative amount of three unsaturated fatty acid in FH and FL. Note: The two-sided *t*-test was used to test the difference significance between FH and FL on the main fatty acids. * indicated the significance at 0.05 probability level.

A total of 33 lipid metabolites were detected in this study, with a retention time from 7.09 to 30.56 min (Table S9). Among them, seven fatty acids had higher concentrations (greater than 0.0001) in both materials. These included four saturated fatty acids, myristic acid, palmitic acid, stearic acid, and arachidic acid, and three unsaturated fatty acids, palmitoleic acid, linoleic acid, and oleic acid. Among them, myristic acid and palmitic acid had the highest content of fatty acid components in rice, while palmitoleic acid had the lowest content. Subsequently, a *t*-test was used to analyze the differences in fatty acid composition between rice grains of the two materials with different taste qualities. The results showed that the relative levels of palmitic acid and oleic acid were significantly higher in the FH material (Figure 8), and this finding may contribute to the superior taste quality observed in the FH material.

## 4. Discussion

The relationship between rice lipid content and grain quality has long been a subject of scientific inquiry, as rice is a staple crop for food security and nutrition. Our study developed 94 recombination inbred lines aimed to explore the intricate connection between rice lipid composition and rice quality. Our investigation unveiled a significant positive correlation between rice lipid content and taste quality. Notably, the materials with a higher lipid content tended to exhibit improved taste quality, and factors such as rice palatability, glossiness, and aroma were notably enhanced in rice grains with higher lipid content. Consequently, elevating lipid content could significantly improve rice fragrance and overall quality, aligning with the findings of previous research [4,6,13,46,47].

Furthermore, amylose content and protein content were also the key factors that had an impact on rice quality [48,49,50,51,52]. And, some studies have suggested a correlation between rice lipid content and other contents. The lipids could affect the hydroxylation of starch, and an increase in lipid content would lead to a decrease in amylose content [53]. However, our study within the RIL population did not reveal a significant correlation between rice lipid content and protein content or amylose content. This discrepancy may arise from the insufficiently significant differences in protein and amylose content between the two parental lines used in creating the RILs. Notably, unsaturated fatty acids within non-starch lipids are susceptible to environmental factors, leading to oxidation and a subsequent decline in rice quality [20,21]. In our study, rice varieties with superior taste quality exhibited higher lipid content. However, it is worth noting that high lipid content did not necessarily correspond to elevated levels of non-starch lipids. The absence of a significant positive correlation between these two variables allowed us to identify rice materials with high lipid content yet moderate non-starch lipid content. This approach provides a means to enhance rice taste quality without compromising its storage stability.

Lipids constitute a crucial component in rice, distinct from starch and proteins, and their synthesis and metabolism processes are intricate. Numerous quantitative trait loci (QTLs) or genes associated with lipid metabolism have been identified or cloned [23,24,25,26,30,31]. To explore the molecular mechanism behind the impact of lipid content on rice taste quality, this study employed two rice materials, FH and FL, which exhibit similar agronomic traits but differ in lipid content. We conducted transcriptome analysis on rice grains during the grain-filling stage, revealing a total of 619 differentially expressed genes. In the context of lipid metabolism, two genes, *Os07g0417200* and *Os12g0102100*, emerged as the key regulators of lipid synthesis in FH materials. *Os07g0417200* (*OsFAD2-1*), which has been previously cloned, encodes a fatty acid desaturase. Previous studies reported that the desaturase genes play an important role in plant growth and development. They are used to synthesize unsaturated fatty acids in many planta, such as rice [31], walnuts [54], rape [55], and soybeans [56]. And, these desaturase genes, especially *FAD2* and *FAD3*, also showed a positive response to abiotic stresses such as cold in rice, wheat, or olives [57,58,59,60]. In this paper, *OsFAD2-1* exhibited multiple variation sites in its genome and was expressed significantly less in the grain-filling period in the FH compared to the FL lines, increasing the synthesize of unsaturated fatty acids, such as oleic acid of grains. A previous study reported that reducing its expression can control the increase in oleic acid and the decrease in linoleic and palmitic acids [31,32]. Our findings verified the pivotal role of desaturase genes in affecting rice lipid content and taste quality.

Additionally, the novel gene *Os12g0102100* encodes an alcohol dehydrogenase and is significantly enriched in various metabolic pathways, including fatty acid biosynthesis, and can influence rice yield [61]. In our study, *Os12g0102100* exhibited the same haplotype in all three materials, with one SNP in the promoter region compared to the reference genome. This variation site potentially leads to a significantly higher expression of this gene in JLX, consequently altering the content of various fatty acid components. Based on these genes affecting fatty acid synthesis known in plants, altering the expression levels of these genes during grain filling by molecular methods could change the fat content or fatty acid composition in rice. Yin et al. used embryo-specific expression of ω3/Δ15-desaturase gene to increase α-linolenic acid content in rice bran [62]. Moreover, the overexpression of *GmFAD3-1* and *OsFAD3* could increase linolenic acid content in seeds [30]. And, gene editing technology could be used to eliminate *OsFAD2-1*, showing that oleic acid content increased by two times compared with the wild type [32].

Furthermore, the lipids may form starch–lipid complexes with long-chain amylopectin to increase the content of resistant starch affecting rice quality [45,46], and the loss function of *OsSSIIIa* [41] *or OsBEIIb* [44] may exacerbate this formation of complexes. In this article, we found that these two genes were up-regulated in the FH material with high fat content. This suggested that increasing the lipid content of seeds did not necessarily increase resistant starch content.

In conclusion, our research demonstrates that fat content plays an important role in rice quality and that there was a positive relationship between fat content and rice taste quality. Furthermore, most genes related to fatty acid synthesis, elongation, or degradation that were discovered displayed significantly different expression between FH and FL lines via transcriptome analysis, resulting in a difference in fat content and taste quality between the two lines. Finally, two genes (*Os07g0417200* and *Os12g0102100*) with a sequence variation might be the key genes to affect the synthesis unsaturated fatty acids, thus leading to the discrepancy of fatty acid components in rice grains. Molecular biology techniques can be employed to manipulate or edit genes to alter the nutritional quality and eating quality of rice in the future. In short, our study indicated the effect of fat content on the eating quality of rice, providing initial insights into the molecular mechanisms of regulating lipid metabolism to improve rice taste quality. Finally, our study provided a theoretical basis for the molecular design and breeding of high-quality rice varieties.

## Figures and Tables

**Table 1:** Nutritional quality of three rice varieties.

Variety	Apparent Amylose Content (%)	Non-Starch Lipids Content (%)	Crude Fat Content (%)	Crude Protein Content (%)
XHZ	18.42 ± 0.31 a	0.19 ± 0.02 a	0.98 ± 0.02 a	7.02 ± 0.21 a
HGLZ	15.48 ± 0.21 c	0.26 ± 0.00 b	1.13 ± 0.03 a	6.21 ± 0.33 b
XYXZ	16.98 ± 0.15 b	0.25 ± 0.01 b	1.73 ± 0.11 b	6.13 ± 0.23 b

Note: Data were expressed as mean ± standard deviation, *n* = 10. The least significant difference (LSD) method is used to compare the means between three materials on rice quality. Different letters within the same column indicate significance at 0.05 probability level.

## Data Availability

The raw data of RNA-seq reads were deposited in the NCBI database under the accession number PRJNA1056928.

## Supplementary Materials

The following supporting information can be downloaded at https://www.mdpi.com/article/10.3390/genes15010081/s1, Table S1: Nutritional quality and taste quality of 94 recombination inbred lines; Table S2: The number of sequencing obtained from each sample and the clean reads mapped to the reference genome; Table S3: All DEG FPKM values among four six lines; Table S4: Analysis of genes regarding differentially expressed starch and sucrose metabolism in FH material compared with FL material; Table S5: Analysis of genes regarding differentially expressed seed storage protein synthesis in FH material compared with FL material; Table S6: Analysis of genes regarding differentially expressed fatty acid metabolism in FH material compared with FL material; Table S7: Analysis of genes regarding differentially expressed phenylpropanoid biosynthesis in FH material compared with FL material; Table S8: Analysis of genes regarding differentially expressed cutin, suberine, and wax biosynthesis in FH material compared with FL material; Table S9: The 33 lipid metabolites detected in FH and FL grains by off-target metabolome; Table S10: Primer sequences used in this paper.
